# Low Vitamin D and Its Association with Cognitive Impairment and Dementia

**DOI:** 10.1155/2020/6097820

**Published:** 2020-04-30

**Authors:** Sadia Sultan, Uzma Taimuri, Shatha Abdulrzzaq Basnan, Waad Khalid Ai-Orabi, Afaf Awadallah, Fatimah Almowald, Amira Hazazi

**Affiliations:** College of Applied Medical Sciences, Umm Al-Qura University, Abdiya, Makkah, Saudi Arabia

## Abstract

Vitamin D is a neurosteroid hormone that regulates neurotransmitters and neurotrophins. It has anti-inflammatory, antioxidant, and neuroprotective properties. It increases neurotrophic factors such as nerve growth factor which further promotes brain health. Moreover, it is also helpful in the prevention of amyloid accumulation and promotes amyloid clearance. Emerging evidence suggests its role in the reduction of Alzheimer's disease hallmarks such as amyloid-beta and phosphorylated tau. Many preclinical studies have supported the hypothesis that vitamin D leads to attentional, behavioral problems and cognitive impairment. Cross-sectional studies have consistently found that vitamin D levels are significantly low in individuals with Alzheimer's disease and cognitive impairment compared to healthy adults. Longitudinal studies and meta-analysis have also exhibited an association of low vitamin D with cognitive impairment and Alzheimer's disease. Despite such evidence, the causal association cannot be sufficiently answered. In contrast to observational studies, findings from interventional studies have produced mixed results on the role of vitamin D supplementation in the prevention and treatment of cognitive impairment and dementia. The biggest issue of the existing RCTs is their small sample size, lack of consensus over the dose, and age of initiation of vitamin D supplements to prevent cognitive impairment. Therefore, there is a need for large double-blind randomized control trials to assess the benefits of vitamin D supplementation in the prevention and treatment of cognitive impairment.

## 1. Background

Vitamin D is a fat-soluble steroid vitamin with a definitive role in bone health. Beyond its role in the regulation of bone health, it also plays an important role in the functioning of other systems such as cardiovascular, endocrine, and nervous systems [1]. Ultraviolet radiation (UVR) is the major source of vitamin D. The two forms of vitamin D are ergocalciferol (D2) and cholecalciferol (D3). It undergoes two hydroxylation processes, first in the liver by enzyme 25 hydroxylase to produce 25(OH)D and second in the kidney to produce active form of 1,25(OH)D [2, 3]. An estimated one billion people worldwide suffer from hypovitaminosis D. There is no worldwide consensus regarding the cutoff value for definition of vitamin D deficiency. Typically, vitamin D deficiency is defined as a 25(OH)D level of less than 50 nmol/L, with severe deficiency defined as less than 25 nmol/L and insufficiency between 50 and 75 nmol/L [4]. Vitamin D can reach the brain by crossing the blood-brain barrier (BBB) through passive diffusion. The active form, 1,25(OH)D, binds to the vitamin D receptor (VDR) and influences gene expression. Vitamin D exerts its action via VDR present in neurons, glial cells of the hippocampus, orbitofrontal-cortex, cingulate, amygdala, and thalamus [5–7]. Its neuroprotective, anti-inflammatory, and antioxidant effect on neurons promotes brain health [8–10]. Vitamin D promotes the production of neurotrophic factors such as nerve growth factor (NGF). Many studies have consistently reported the increase in neuronal growth in rat hippocampal cell cultures enriched with vitamin D [8, 9]. The NGF and other neurotrophic factors promote the survival of both hippocampal and cortical neurons [10, 11]. Vitamin D is also implicated in regulating the gene expression of various neurotransmitters such as acetylcholine, dopamine serotonin, and gamma butyric acid [12, 13]. Vitamin D reduces age-related tau hyperphosphorylation, the formation of amyloid-beta oligomers, increases amyloid clearance, and prevents neuronal death [13]. Although it promotes amyloid phagocytosis and clearance, correlation of serum vitamin D with CSF (cerebrospinal fluid) biomarkers of amyloidosis such as phosphorylated tau and amyloid-beta is, by far, not investigated except very few studies [13]. Nevertheless, vitamin D has also shown its neuroprotective activity by curtailing the glutamate-induced neurotoxicity [13] and upregulating genetic expressing of various proteins required for new synapse formation, thus promoting neurogenesis especially in the hippocampus [14]. Neuroimaging has suggested a positive association between low vitamin D levels, white matter hyperintensities, and enlarged frontal horn of the lateral ventricle [15]. Significant positive correlation of serum 25(OH)D with total hippocampus volume and disrupted structural connectivity between hippocampus, cortical, and subcortical areas in the right hemisphere was found in patients with mild cognitive impairment [16]. However, small sample size, cross-sectional design, and lack of detailed data on potential covariates (hypertension and diabetes) were the limitations of the study. Furthermore, prospective studies of longer duration exploring neuroimaging outcomes will provide useful insights into potential mechanisms as most neuroimaging studies have been cross-sectional resulting in the possibility of reverse causation. This review aims to provide an overview and discussion of the current state of evidence regarding vitamin D and dementia-related outcomes.

## 2. Vitamin D and Brain-Evidence through Animal Studies

Developmental vitamin D deficiency and inactivated vitamin D receptor gene affect brain functioning and behavioral outcome in rodents. The studies which support this hypothesis were conducted on mice with prenatal deficiency of vitamin D and vitamin D knock-out mice. Rats born to vitamin D3-deficient mothers demonstrated a reduction in the nerve growth factor and glial-derived neurotrophic factor compared to control rats [17]. Similarly, a study on 10-week-old rats with transient vitamin D deficiency during the early developmental stage demonstrated enlarged lateral ventricle volume and reduced nerve growth factor compared to controls [18]. The evidence concerning the impact of vitamin D deficiency on the behavior of mice which developed later in life is sparse. However, a study investigating the effect of vitamin D-deficient diet for 10 weeks on 20-week-old mice reported behavioral and neurochemical changes [19]. Similarly, another study reported a subtle effect on attentional tasks in 16–20-week-old rats with a vitamin D-deficient diet given for 10 weeks compared to control rats [20].

### 2.1. Evidence through Cross-Sectional and Longitudinal Studies

The association of low vitamin D and global cognitive deficit is established through many cross-sectional and longitudinal studies. Nevertheless, the issue of reverse causality remains to be answered [21].  Table 1 shows a summary of evidence demonstrating an association between serum 25(OH)D, CI, and dementia [9, 21, 22, 23–26]. Many studies have shown an association of low vitamin D with CI at a cross-sectional level although the same studies with longitudinal follow-ups did not replicate the association [21, 23, 24]. All included studies showed a difference in the study population, sample size, participants' age, follow-up time, vitamin D exposure, method used for estimation for vitamin D, criteria used to diagnose dementia and CI, and methods of assessment of cognition. Most studies adjusted for confounders like age, education, physical activity, diabetes, hypertension, hypercholesteremia, and season. However, most studies did not consider confounders like depression. Four studies [9, 22, 23, 25] found no association between low serum vitamin D levels and CI and dementia in a longitudinal follow-up, whereas two studies [21, 24] found a significant association (*P*=0.001). However, the studies which found a significant association were smaller in size and duration of follow-up. A Swedish study [22] done on a large sample (2,841) for a longer follow-up (18 years) did not find an association. This study took into account the confounders like common dietary intake of vitamin D, physical activity, and sun exposure. However, repeated blood sampling and dietary assessments improve the precision of exposure information, the study lacked in doing so. Similarly, another American study [25] done on a large sample (13,044) with a long follow-up (20 years) did not report any such association. The previously reported associations between 25(OH)D concentrations and cognitive impairment may be a result of reverse causation—whereby low 25(OH)D is a marker of poor health (resulting from those in poor health (e.g., those with cognitive impairment) doing less physical activity and having less sun exposure and thereby having lower vitamin D concentrations) rather than a causative factor in cognitive impairment and dementia pathogenesis. This study can be considered less susceptible to reverse causation as 25(OH)D was measured in midlife, and cognitive change was evaluated over 20 years. Another methodological shortcoming compromising the validity of the data is the use of single serum 25(OH)D measurements taken at baseline to represent long-term exposure in all studies [9, 21, 22, 23–25]. A prospective study with two follow-ups, each at 5 years, conducted to examine the association of dietary and supplemental vitamin D intake and cognitive decline showed an association between high intake and a slower decline in the cognitive domains of verbal fluency. Those with supplemental intake also exhibited a slower decline in the cognitive domain of verbal fluency although the effect on visual and verbal memory was less in magnitude [26]. Similarly, a study on participants (age 55–67 years) with levels >25 nmol/l has demonstrated better verbal fluency and executive functioning both at baseline and at a 10-year follow-up [27].

In contrast to the existing body of literature demonstrating a positive correlation between cognitive function and vitamin D status, Lam et al. [28] reported a negative association between vitamin D levels and verbal episodic memory. In a prospective (3-year follow-up), population-based study of older adults aged 85+, it was found that both low and high season-specific quartiles of 25(OH)D were associated with higher odds of prevalent cognitive impairment (assessed by MMSE), poorer attention reaction times/processing speed and focused attention/concentration, and greater attention fluctuation [29].

### 2.2. Evidence through Meta-Analysis and Systematic Review

Several systematic reviews and meta-analysis of cross-sectional studies, case-control studies, and observation prospective studies have suggested an association between low vitamin D, cognitive impairment, and dementia. Moreover, a meta-analysis on vitamin D levels and specific cognitive domains have suggested a strong association between low vitamin D and a range of executive dysfunction, such as impaired processing speed, mental shifting, and information updating. Only a modest association was noted with episodic memory [30]. Several such systematic reviews and meta-analysis in the last 6 years are depicted in Table 2 [30–34].

### 2.3. Vitamin D Supplementation and Cognition

Five studies have investigated the effects of vitamin D supplementation on cognitive outcomes in elderly individuals (see Table 3); three were RCT's [36–38] and two had pre-post study design [8, 37]. Overall, three studies found that vitamin D supplementation did not improve either cognitive outcomes [36, 38, 39] or reduce the risk of dementia/MCI compared to controls. A prospective pre-post interventional study [39] on nursing home residents with a mean age (86 years) reported no significant change in cognitive outcome with oral vitamin D2 (50,000 IU 3 times/week) for 4 weeks. On the contrary, another prospective pre-post interventional study [37], which included 80-year-old subjects from memory clinic, found that those who received oral vitamin D3 supplementation (800 IU per day or 100,000 IU per month) experienced improved global cognition and executive functioning abilities over a 16-month follow-up period compared to controls [40]. Nevertheless, the pre-post design (without randomization) of the study and small sample size and shorter duration of treatment limit the exploration of cognitive effect of vitamin D. A randomized trial [38] found that visual memory improved in the high dose group (4,000 IU per day for 18 weeks of oral vitamin D supplementation) when compared to the low dose group (400 IU per day) in healthy adults, although verbal memory and other cognitive domains did not improve. On the contrary, Stien et al. [36] reported no significant change in cognition with higher doses of vitamin D followed by intranasal insulin (nasal insulin improves cognition, and vitamin D increases insulin receptor expression) when compared to lower dose of vitamin D and intranasal insulin in subjects diagnosed with mild-to-moderate AD. A more recent double-blind, randomized, placebo-controlled trial showed no significant difference in cognition over time (3 years) according to the MMSE score (assessed every 6 months) between older postmenopausal African-American women who took vitamin D (orally in doses of 2,400, 3,600, and 4,800 which maintained serum level of >30 ng/mL) than those who did not [37]. However, methodological weaknesses such as small sample sizes [36, 38, 39], short follow-up periods [36, 38, 39], and lack of participant randomization [39, 40], as well as heterogeneous doses of vitamin D supplementation and baseline vitamin D levels make it difficult to interpret the results of the interventional studies. Another limitation found in most studies was the use of MMSE for cognitive testing. This test is best used as a screening tool and not for diagnosis. There is no clear idea of when vitamin D is most effective in the pathogenesis of cognitive decline and particularly the advent of AD. Therefore, the supplementation of vitamin D after the advent of CI or AD might not have helped the already existing neurological insult which could have been the reason for the failure of such a treatment. Larger trials over a longer period in patients at risk for, but has not yet progressed to cognitive decline or dementia, may be more capable of demonstrating an impact. Identifying such individuals using CSF biomarkers such as amyloid-beta and phosphorylated tau may help. Future studies directed towards finding the effect of vitamin D on biomarkers of AD would further clarify the role of vitamin D and its disease-modifying effect. Pharmacogenomic studies to identify the individuals who could benefit from such a therapy may further help.

## 3. Conclusion

Evidence from animal and cellular studies suggests that vitamin D has multiple functions throughout the central nervous system and could be implicated in the prevention and treatment of disorders such as dementia and AD. Cross-sectional and case-control studies confirm that vitamin D concentrations are lower in individuals with cognitive impairment and dementia although reverse causality remains a possibility. Few longitudinal studies have found that low vitamin D concentrations are associated with an increased risk of cognitive decline, all-cause dementia, and AD, but those with a bigger sample size and longer (18–20 years) follow-up time did not find such an association. Future neuroimaging studies may uncover a link with specific abnormalities that could explain the observed associations between vitamin D concentrations and dementia-related disorders. Clinical trials investigating the effect of vitamin D supplementation on cognitive outcomes have produced mixed findings; however, a variety of methodological weaknesses limit the interpretability of these findings. Lack of consensus over the exact dosage of vitamin D to be used and optimal age of treatment initiation of individuals at risk remains unidentified. Furthermore, large double-blind, randomized, placebo-controlled trials with appropriate dosage and duration may provide conclusive results. Taken together, this body of evidence suggests that vitamin D may be a new paradigm for therapy in the prevention and treatment of dementia and AD. Although vitamin D may be considered as a modifiable risk factor, the causal relationship between vitamin D deficiency and CI so far remains inconclusive.

## Figures and Tables

**Table 1:** Summary of cross-sectional and longitudinal studies depicting association between serum 25(OH)D, cognitive impairment, and Alzheimer's disease and other dementia.

Study	Study design	Sample size	Study period	Population	Cognitive test	Vitamin D levels	Outcomes
Schneider et al. [25]	Longitudinal study	13,044	20 years	Mean age 57 years From USA	DWRT DSST, WFT	Insufficiency 20–30 ng/ml Sufficiency > 30 ng/ml Deficiency < 20 ng/ml	Multivariate-adjusted linear mixed effect model used for analysis. No significant association with cognitive decline deficient versus sufficient: –0.035 (95% CI – 0.104 to 0.033) and intermediate versus sufficient: –0.029 (95% CI – 0.080 to 0.023)).

Olsson et al. [22]	Longitudinal study	2,841	18 years	Mean age 68 years From Sweden	MMSE	Deficient < 50 nmol/L Sufficient > 75 nmol/L	Cox proportional hazards regression (95% CI: 0.59, 1.31) in men with plasma 25(OH)D concentrations 50 compared with 0.75 n·mol/L. (95% CI: 0.63, 1.32) for the lowest compared with highest tertiles of vitamin D intake.

Feart et al. [21]	Longitudinal study	916	12 years	Mean age 65 years and more From France	MMSE Benton visual retention test Trail making test	Sufficient > 50 nmol/L Insufficient 25–50 nmol/L Deficiency < 25 nmol/L	Multivariate analysis Vitamin D deficiency and insuffiency had double the risk of all cause dementia with 95% CI (1.21–3.71) for deficiency and 95% CI (1.17–3.36) for insufficiency

Beydoun et al. [26]	Longitudinal study	1,803	10 years	Age (30–64 years) From USA White urban adults African-Americans	MMSE CVLT TMT-B BVRT CDT DF-S and DFS-B AF		Linear regression Higher aseline serum 25OHD was linked toa slower decline in verbal fluency (*p* < 0.001) Higher intake of vitamin D was associated with aslower rate of decline constructive ability (*p* < 0.001). Use of vitamin D supplements during follow-up was related to slower rate of decline in verbal fluency among older individuals.

Goodwill et al. [27]	Longitudinal study	252	10 years	Age (55–67 years) From Australia	CVLT-11 TMT-B CERAD,	Deficient < 25 nmol/L	One-way ANOVA and Pearson Chi square Vitamin D > 25n·mol/L performed better on verbal fluency (95% CI = 0.53, 4.40) and TMT-B time (95% CI = −32.86, −3.61), with higher executive function (95% CI: 0.44,2.37) These relationships persisted 10 years later in the follow-up

Laughlin et al. [9]	Longitudinal study	1,058	12 years	MMSE TMT Halstead–Reitan neuropsychological test Animal naming category test, fluency test	MMSE TMT Halstead–Reitan neuropsychological test Animal naming category test, fluency test	Insufficiency ˂ 30 ng/ml	Linear mixed effect regression model. Vitamin D insufficiency associated with poor performance on MMSE(*p*=0.013). No association found on follow-up

Jorde et al. [23]	Longitudinal study	4,624	13 years	Age 54.9 years From Norway	MMSE	Continuous variable	Linear regression Association of low vitamin D and CI found in older, more than 65 years (*p*=0.001) at cross-sectional level; no association found on follow-up

Moon et al. [24]	Longitudinal study	412	5 years	Mean age 74 From Korea	MMSE	Deficiency 25–49 nmol/L Severe deficiency ˂ 25nmol/L Sufficiency ˃ 50 nmol/L	One-way ANOVA Severe Vitamin D deficiency was associated with future risk of MCI and dementia. 95% CI (1.46–14.8)

Lam et al. [28]	Longitudinal study	179	—	Mean age 74 From Korea	MMSE and RAVLT	31-334.4 mol/L (mean 84.7 nmol/L)	Bayesian mixed model 25(OH)D levels were negatively associated

MSE: Mini-Mental Status Examination, WFT: word fluency test, DSST: digit symbol substitution test, DWRT: delayed word recall test, and TMT: trial making test; MCI: mild cognitive impairment and RAVLT: Rey auditory verbal learning test; Consortium to Establish a Registry for Alzheimer's Disease (CERAD), California Verbal Learning Test Second Edition (CVLT-II), verbal fluency and trail making test-B (TMT-B), delayed free recall (DFR), digit span forward and backward tests (DS-F and DS-B), Benton Visual Retention Test (BVRT), animal fluency test (AF), Brief Test of Attention (BTA), and Clock Drawing Test (CDT).

**Table 2:** Overview of systematic reviews with or without meta-analysis depicting the association of serum 25(OH)D with cognitive impairment and Alzheimer's disease and other dementia.

First author, year	Inclusion criteria	Outcomes	Assessment of dementia	Number of articles included	Number of patients	Design of the included articles	Main conclusion
Goodwill, 2017 [31]	Longitudinal, cross-sectional, and interventional studies. Data of serum 25OHD in healthy adults and its correlation with CI and dementia	Dementia and MCI	Cognitive function tests (e.g., MMSE, Boston naming test, Stroop test, Raven's progressive matrices, clock drawing; block)	41 studies	9,556	Cross-sectional (*n* = 20) Longitudinal (*n* = 18) Interventional (*n* = 3)	Significant association in cross-sectional studies (95% CI I1.09 to 1.23) and weak association in longitudinal studies. No benefit with vitamin D supplementation (95% CI −0.05 to 0.46)

Sommer, 2017 [3]	Prospective and retrospective studies with data on serum vitamin D and dementia	Dementia: Alzheimer's disease vascular, frontotemporal, Lewy body	Diagnostic criteria (e.g., ICD10, DSM4, NINCDS-ADRDA)	6 studies	18,933	Prospective (*n* = 5), Retrospective (*n* = 1)	Low evidence of vitamin D deficiency increasing the risk of dementia (95% CI 1.19 to 1.99)

Kuzma, 2016 [32]	Prospective studies with data on serum 25OHD and subsequent development of dementia, visual and verbal memory loss	Dementia, Visual and verbal memory	Diagnostic criteria (e.g., NINCDS-ADRDA criteria). Cognitive function tests (MMSE, Benton visual retention test, Reys auditory verbal learning test)	2 studies	1,291	Prospective studies (*n* = 2)	Those moderately and severely deficient individuals with serum 25(OH)D changed (95% CI: −0.06 to 0.01) and (95% CI: −0.19 to −0.02) per year, respectively, in visual memory compared to those sufficient serum 25(OH)D were associated with a mean change of 0.01 SD (95% CI: −0.01 to 0.02) and (95% CI: −0.04 to 0.02) per year, respectively, in verbal memory compared to sufficiency

Shen, 2015 [33]	Data on 25(OH)D concentration and Alzheimer's disease or dementia	Alzheimer's disease and dementia	Diagnostic criteria (e.g., ICD-10, DSM- 4NINCDS-ADRDA criteria)	Alzheimer's disease: 5 studies, 5 studies Dementia: 5 studies, 4 studies	Alzheimer's disease: 10,019 Dementia: 5,073	Alzheimer's disease: cross-sectional (*n* = 1), prospective (*n* = 4) Dementia: cross-sectional (*n* = 3), prospective (*n* = 2)	Lower 25(OH)D status is associated with increased risk of developing AD and dementia (OR = 1.63, 95% CI 1.01–1.40)

Lopes da Silva, 2014 [34]	Data on any type of plasma nutrient status and Alzheimer's disease	Alzheimer's disease	Diagnostic criteria (e.g., NINCDS-ADRDA, DSM III or IV)	5 studies	865	Case-control (*n* = 5)	No association between low levels of 25(OH)D and Alzheimer's disease (95% CI −12.11 to 0.58)

Annweiler, 2013 [30]	Any type of observational study, data on 25(OH)D and cognition	Memory and executive dysfunction	Cognitive function tests (e.g., word list recall, serial digit recall frontal assessment battery, TMT, DST)	17 studies	39,975	Cross-sectional (*n* = 11), prospective (*n* = 1)	Low serum 25(OH)D concentration found in AD when compared to controls. No association found in longitudinal study and inconsistencies in results seen with 3 case control studies (95% CI 0.26–2.56)

van der Schaft, 2013 [35]	Observational studies with data on vitamin D (serum concentration or dietary intake and cognition)	Cognition	Cognitive function tests (e.g., MMSE, TMT, DST, n-back test, block design test)	8 studies	59,576	Cross-sectional (*n* = 25), prospective (*n* = 6)	Low serum 25(OH)D was associated with higher frequency of dementia on follow-up of 4–7 years

NINCDS-ADRDA: National Institute Neurological and Communicative Disorders and Stroke-Alzheimer Disease and Related Disorders Association, ICD-10: International Classification of Disease, DSM4: Diagnostic and Statistical Manual for Mental Diseases, CDR: clinical dementia rating, MCI: mild cognitive impairment, MMSE: Mini-Mental Status Examination, TMT: trial making test, CI: cognitive impairment, AD: Alzheimer's disease, and DST: digit symbol test.

**Table 3:** Summary of findings of vitamin D supplementation and its effect on cognition.

Author, year	Study design	Sample size	Study period	Population characteristics	Intervention	Outcome measures	Findings
Przybelski et al., 2008 [39]	Prospective Pre-post interventional study	63	4 weeks	USA-nursing home residents Intervention groups *N* = 25 Mean age = 86.2 females 68% Deficiency defined as 25(OH)D ˂25 ng/mL Comparison group *N* = 38 Mean age = 87.4, female 78.9% Serum 25(OH)D ˂25 ng/mL	Unblinded study Intervention group 50,000 IU of Vit D2, 3 times per week for duration of 4 weeks Comparison group No placebo No supplementation	Cognitive test Clock drawing test Semantic fluency test	No significant difference in cognitive outcome measures, although significant change (*p*=0.0001) in serum 25(OH)D levels from 17.3 to 63.8 ng/mL

Stein, et al., 2011 [36]	Randomized controlled trial	32	6 weeks	Australia- community dwellers with mild-to-moderate dementia High dose group *N* = 16 Low dose group *N* = 16 Mean age 79 Females = 50%	First 1,000 IU Vit D2 daily for 8 weeks Then, 6,000 IU Vit D2 daily for 8 Intranasal insulin (60 IU/4 times/day) and 50% randomized to placebo Comparison group 1,000 IU Vit D2 daily for 16 weeks Finally, 50% randomized to Intranasal insulin (60 IU4 times/day) and 50% randomized to placebo for 2 days	Alzheimer assessment scale-cognitive subscale (ADAS memory scale-revised logical memory (WSM- RLM)	Minitab release 13.1 used to calculate CI. Significant median increase in serum 25(OH)D concentrations in the high dose group (187 nM) No significant differences (*p*=0.02) on any of the outcome measures) between groups. ADAS-Cog (95% CI-5 to 3) WSM-RLM (95% CI −1 to 3)

Annweiler, et al., 2012 [40]	Prospective Pre-post interventiona l study	44	16 months	France-outpatient from memory clinic Intervention group *N* = 20 Mean age 81.9 females = 55% Comparison group *N* = 24 Mean age 75. Females = 54.2%	Unblinded Unrandomized intervention group = 800 IU/day or 1,000,000 IU of Vit D3/month comparison group = no placebo	MMSE Frontal assessment battery Cognitive assessment battery	Over the 3-year period, MMSE scores increased in both groups (*p* < 0.001), although change over time was not significantly different between the groups

Owusu et al., 2018 [37]	Randomized controlled trial	390	3 years	USA-healthy African-American postmenopausal women Intervention group *N* = 130 Mean age 67.8 Control group *N* = 130	Double-blind randomized placebo-controlled trial Intervention Vit D3 (2,400 IU–3,600 IU or 4,800 IU/day) which maintained 25(OH)D level of 30 ng/mL Control group Placebo follow-up of 3 years	MMSE Every 6 months ˂ 27 Considered as mild MCI	No difference in cognition over time between older African-American women with serum concentrations of 25(OH)D of 30 ng/mL and greater than those taking placebo

Petterson Trial, 2017 [38]	Randomized controlled trial	82	18 weeks	Canada-healthy adults High dose group *N* = 42 Mean age = 56.7 Low dose group *N* = 40 Mean age = 52.6 Females = 65%	Randomized and blinded to high dose High dose group Vit D3 4,000 IU/day Low Dose group Vit D3 400 IU/day	Pattern recognition memory task Paired associate learning task	Visual memory benefit with high dose (*p*=0.005) in those who are insufficient (<75 nmol/L) No change in verbal memory

## Abbreviations

25(OH)D: 25 hydroxyvitamin D
A*β*: Amyloid-beta
AD: Alzheimer's disease
AMSTAR: Assessing the methodological quality of systematic reviews
BBB: Blood-brain barrier
BDNF: Brain-derived neurotrophic factor
CDR: Clinical dementia rating
CI: Cognitive impairment
CKD: Chronic kidney disease
CSF: Cerebral spinal fluid
DSM4: Diagnostic and Statistical Manual for Mental Disease
DSST: Digit symbol substitution test
DST: Digit symbol test
DWRT: Delayed word recall test
GM: Grey matter
HPV: Hippocampal volume
ICD: International Classification of Disease
ICV: Intracranial volumes
IOM: Institute of medicine
LTP: Long-term potentiation
MCI: Mild cognitive impairment
MMSE: Mini-Mental Status Examination
NGF: Nerve growth factor
NINCDS-ADRDA: National institute neurological and communicative disorders and stroke-Alzheimer disease and related disorders
NMDA: N-Methyl d-aspartate
RCT: Randomized controlled trials
RDA: Recommended daily allowances
TMT: Trial making test
UVR: Ultraviolet radiation
VDR: Vitamin D receptor
WFT: Word fluency test
WM: White matter
WMA: White matter abnormalities
WMH: White matter hyperintensities.
